# A scoping review of authorisation pathway for COVID**-**19 vaccines among selected countries

**DOI:** 10.1080/20523211.2025.2520861

**Published:** 2025-07-02

**Authors:** Supapitch Suphap, Parnnaphat Luksameesate, Osot Nerapusee, Puree Anantachoti

**Affiliations:** a Medicines Regulation Division, Thailand Food and Drug Administration, Ministry of Public Health, Nonthaburi, Thailand; b Faculty of Pharmaceutical Sciences, Graduate Program in Social and Administrative Pharmacy, Chulalongkorn University, Bangkok, Thailand; c Faculty of Pharmaceutical Sciences, Department of Social and Administrative Pharmacy, Chulalongkorn University, Bangkok, Thailand; d School of Pharmacy, Eastern Asia University, Pathumthani, Thailand

**Keywords:** COVID-19 vaccines, authorisation pathway, approval process, regulation, pandemic

## Abstract

**Background:**

In response to COVID-19 pandemic, NRAs implemented expedited mechanisms such as EUAs and CMAs, relying on international bodies, to authorize vaccines. Documenting these authorization pathways across countries informs NRAs and strengthens future pandemic responses. Existing studies on COVID-19 vaccine approvals are limited, underscoring the need for a comprehensive review. This study aimed to explore approval pathways, investigate supporting strategies, and review times for COVID-19 vaccines.

**Methods:**

A scoping review was conducted, covering countries with stringent regulatory authorities or countries with significant COVID-19 vaccine manufacturing or rapid rollout. Searches using terms such as ‘approval process’ and ‘COVID-19 vaccine’ were performed in electronic databases and on official regulatory authority websites. Countries without authorization information or with language barriers were excluded. Data on vaccine authorization processes including supporting strategies, submission criteria, and approval timelines were extracted and analyzed using content and comparative regulatory analysis.

**Results:**

Twenty-four regulatory pathways from 20 countries were identified, categorized as ‘emergency use authorization’ (EUA) (*n* = 10), ‘conditional marketing authorization’ (CMA) (*n* = 8), and “temporary authorization’ (TA) (*n* = 6). Twelve new pathways were created by 11 countries, with four countries using two pathways each for different pandemic stages. Common requirements for vaccine approval included good manufacturing practice (GMP) (92%), benefit-risk evaluation (86%), and unmet need (83%). Supporting strategies like ‘rolling submission’ (85%) and ‘pre-submission meeting’ (85%) facilitated and fasten the approval process. Non-high-income countries favored the ‘reliance’ strategy, while high-income countries engaged in regulatory networking. The FDA and EMA were the most referenced agencies. Review times for the first COVID-19 vaccines ranged from 10 to 84 days, significantly faster (3-27 times) than standard review times.

**Conclusion:**

Despite different regulatory pathways, all countries approved the first COVID-19 vaccine quickly. International collaboration was crucial in accelerating approval times. The COVID-19 pandemic positively stimulated global preparedness for serious pandemics.

## Background

COVID-19, caused by severe acute respiratory syndrome coronavirus 2 (SARS-CoV-2), emerged in December 2019 (Zhu et al., 2020). By March 2022, the World Health Organization (WHO) reported 6.15 million deaths and 485.95 million infected cases (World Health Organization, 2022b). Despite measures like social distancing, hand hygiene, and wearing masks (Ayouni et al., 2021), the virus continued to spread relentlessly.

COVID-19 vaccines play a crucial role in safeguarding the public, alleviating healthcare pressure, preventing system breakdown, and mitigating economic turmoil. Rapid marketing authorisation of the vaccine is crucial to prevent the extensive spread of COVID-19.

Normally, vaccine development spans 10–15 years (Kashte et al., 2021), and the marketing authorisation takes additional 18–24 months (Dellepiane & Pagliusi, 2018; Kashte et al., 2021). This regular process is not viable during a global pandemic.

As researchers and pharmaceutical companies raced to develop COVID-19 vaccines, the Centre for Innovation in Regulatory Science (CIRS) conducted a 2020 study on regulatory pathways. Seven major national regulatory authorities (NRAs) from the United States of America (USA), Canada (CAN), Australia (AUS), Japan (JAP), Switzerland (CHE), China (CHN), and European Medicine Agency (EMA) were included (Centre for Innovation in Regulatory Science, 2020), revealing eight unique pathways and four strategies. Four pathways were tailored for COVID-19: Canada’s ‘Interim Order’, USA’s ‘Corona Virus Treatment Accelerated program’ (Administation., 2021), Australia’s ‘COVID-19 Emergency Exemption 2020’ (Therapeutic Goods Administration, 2020), and Switzerland’s ‘Ordinance on Measures to combat COVID-19.’ Interim Order and Ordinance covered vaccines, medicines, and medical devices, while the other two pathways focused on medical devices.

Other existing regulatory pathways that could be employed for the COVID-19 vaccines included ‘priority review’, ‘conditional approval’, ‘expanded access program,’ and ‘EUA’ (BOX 1).

Box 1. **Summarisation of regulatory pathways potentially applicable for marketing authorisation during global pandemic.**

***Priority review***

Existing in seven NRAs, it shortens the reviewing time but not product development time. The marketing authorisation holders (MAHs) must submit all required documents for consideration. It is called ‘accelerated assessment’ in the EU and ‘fast track’ in Switzerland.

***Conditional approval***

Used in the EU, Canada, USA, Australia, and Switzerland, this pathway allows NRAs to authorise products based on limited clinical data, with further information required within a specified timeframe. It is known as ‘conditional approval’ in the EU and China, ‘notice of compliance with conditions’ in Canada, ‘expedited approval’ in the USA, ‘provisional approval’ in Australia, and ‘temporary authorization’ in Switzerland (Franco et al., 2023).

***Expanded access programme (EAP)***

Available in the EU, Canada, USA, Australia, and Japan. it facilitates the use of investigational products under conditions different from those specified in clinical trials. It is also called ‘Special Access Program’ in the USA and ‘Special Access Scheme’ in Australia.

***Emergency Use Approval (EUA)***

Existing in the USA, this pathway applies under emergency circumstances declared by the Department of Homeland Security, facilitating drug availability before market approval.

In 2021, most therapeutic medicinal products in the USA including remdesivir, convalescent plasma, propofol, hydroxychloroquine, chloroquine, bamlanivimab, baricitinib, and casirivimab plus imdevimab were granted through the EUA pathway, whereas only ruxolitinib was approved under the EAP. EUA was utilised more often than EAP because of the scale of the situation, as well as less document requirement that permitted faster approval time (Rizk et al., 2021).

Apart from previously mentioned regulatory pathways, COVID-19 products could use strategies like breakthrough designation, rolling submission, reliance, and recognition. Breakthrough status, used in the EU, USA and China, offers priority review or conditional approval which help speeding the review process**.**

Rolling submission, widely adopted in the EU, USA, Switzerland, and China, enables gradual information submission by MAHs instead of a full dossier. This approach can be combined with conditional approval and emergency use pathways.

Other strategies commonly used in many countries are reliance and recognition. The reliance strategy involves NRAs identifying reference countries whose assessment reports are significantly weighted in the approval decision, reducing review and approval time. Recognition, on the other hand, means the NRA fully adopts the approved decisions of other reliable and designated NRAs. Many Latin American countries authorised COVID-19 vaccines using reliance strategy. The approval times of these countries were much shorter than those in stringent NRAs (van der Zee et al., 2022).

As of December 2022, 13 COVID-19 vaccines had been approved and distributed globally (European Medicine Agency [EMA], n.d.)*. COVID-*19 *vaccines*; U.S Food and Drug Administation, 2022; World Health Organization, 2022a). However, comprehensive information about the diverse regulatory pathways employed and their effectiveness remained absent. The primary objective of this study was to investigate and enhance the health community’s understanding of the regulatory approval processes applied for COVID-19 vaccines across various countries during the pandemic. A secondary aim was to explore supportive strategies and review timelines.

## Methods

A scoping review was conducted to explore the authorisation pathways for COVID-19 vaccines during the pandemic and was reported in accordance with the PRISMA-ScR (Preferred Reporting Items for Systematic Reviews and Meta-Analyses extension for Scoping Reviews) checklist (Tricco et al., 2018 The country selection was based on three main criteria:

1. Countries with Stringent Regulatory Authorities (SRAs) (World Health Organization, 2023), or
2. Countries with vaccine research and development (R&D) or manufacturing capacity, or (3) Countries with rapid COVID-19 vaccine rollout (before March 2021).

A list of selected countries based on the three criteria is shown in Table 1. The PCC framework (Population, Concept, and Context) was used to develop the objectives and eligibility criteria for this scoping review, where P = COVID-19 vaccines, C = authorisation pathway, and C = selected National Regulatory Authorities (Peters et al., 2020).

**Table 1.** List of countries selected by key inclusion criteria.

Country	Selection criteria			NRA’s name	NRA’s website
	Stringent NRAs	Vaccine R&D/ MFD	Rapid vaccine rollout		
Australia (AUS)	✓	✓		Therapeutic Goods Administration	www.tga.gov.au
Brazil (BRA)		✓		Agência Nacional de Vigilância Sanitária	www.gov.br/anvisa
Canada (CAN)	✓	✓		Health Canada	www.canada.ca/en/health-canada.html
Switzerland (CHE)	✓	✓		Swissmedic	www.swissmedic.ch
Chili (CHL)			✓	Instituto de Salud Pública de Chile	www.ispch.cl
China (CHN)		✓	✓	National Medical Products Administration	www.nmpa.gov.cn
Germany (DEU)	✓	✓		Federal Institute for Drugs and Medical Devices	www.bfarm.de
Spain (ESP)	✓			Spanish Agency of Medicines & Medical Devices	www.aemps.gob.es
European Union (EU)	✓			European Medicines Agency	www.ema.europa.eu
United Kingdom (GBR)	✓	✓	✓	Medicines & Healthcare products Regulatory Agency	www.gov.uk/government/organisations/mhra
Indonesia (IDN)		✓		Badan Pengawas Obat dan Makanan	www.pom.go.id
India (IND)		✓		Central Drugs Standard Control Organisation	cdsco.gov.in
Israel (ISR)		✓	✓	Ministry of Health – Pharmaceutical Division	www.health.gov.il/English
Japan (JPN)	✓	✓		Pharmaceuticals and Medical Devices Agency	www.pmda.go.jp/english
South Korea (KOR)		✓		Ministry of Food and Drug Safety	www.mfds.go.kr/eng
The Philippines (PHP)			✓	Food and Drug Administration	www.fda.gov.ph
Russia (RUS)		✓		Ministry of Health of the Russian Federation	www.rosminzdrav.ru
Singapore (SGP)			✓	Health Sciences Authority	www.hsa.gov.sg
Thailand (THA)		✓		Thai Food and Drug Administration	www.fda.moph.go.th
Taiwan (TWN)		✓		Taiwan Food and Drug Administration	www.fda.gov.tw
United States of America (USA)	✓	✓	✓	U.S. Food and Drug Administration	www.fda.gov

### Search strategy and study selection

For this scoping review, our primary sources included peer-reviewed databases from PubMed, Scopus, and ScienceDirect. However, due to the limited availability of published literature during the early stages of the pandemic, together with limited information on the detailed process of marketing authorisation, we also complemented our search with Google Scholar, which indexed scholarly articles, government reports, and policy documents. In addition, we retrieved data directly from the official websites of NRAs of the selected countries. In cases where the NRA websites did not have a user-friendly search function, we used Google’s advanced search operators (e.g. ‘site:.gov’) to locate specific regulatory documents available on those official websites. The literature search included data published through December 31, 2022.

Database searches were performed in PubMed, Scopus, ScienceDirect, and Google Scholar using the following keywords: ((‘Marketing Authorization’ OR ‘Regulatory approval’ OR ‘approval’) AND (‘COVID-19 vaccine’ OR ‘SARS-CoV-2 vaccine’)). For NRAs official websites, we used targeted keywords such as ‘Marketing authorization or approval’ and ‘COVID-19 vaccine or specific vaccine’s name’. In cases where the website did not support advanced search functions, we used Google to help locate specific pages or documents within the NRA’s domain (e.g. site:gov.sg or site:fda.go.th). Please see the detailed search strategy in Supplemental Material – Appendix.

The eligibility criteria included full-text article, official document, or presentation from government organisation or NRAs. In addition, the content must be relevant to COVID-19 vaccine approval, including information on approval pathways, decision criteria, decision-making processes, and approval timelines. Only documents available in English or Thai were included.

For the data searching process, SS and PL divided the task of searching across different databases and official websites of NRAs using a predefined set of keywords. After removing duplicates, SS and PL independently screened the titles and abstracts of the retrieved records. In cases of disagreement during the study selection process, ON and PA were consulted to discuss and resolve the issue through consensus.

### Data extraction

SS and PL independently extracted the relevant data from the selected documents using a standardised data extraction form developed specifically for this study, which was adapted from previous studies (Saint-Raymond et al., 2020). In cases where inconsistencies arose, the issues were reviewed and discussed with ON and PA to reach a mutually agreed conclusion.

The key information extracted from the selected documents included: (i) marketing authorisation details such as the regulatory pathways, objectives, submission criteria, supportive regulatory strategies, and approval timelines; and (ii) country-specific information such as characteristics of the NRAs, and relevant laws and regulations related to COVID-19.

### Data analysis

We employed both content analysis and comparative regulatory analysis. Content analysis was used to systematically organise and categorise data by country based on aspects such as regulatory pathways, objectives, submission criteria, supportive regulatory strategies, and approval timelines. Subsequently, a comparative regulatory analysis was conducted to identify patterns, similarities, and differences in regulatory approaches across countries. This dual-method approach allowed us to develop a comprehensive understanding of how COVID-19 vaccine approvals were managed across jurisdictions.

### Results

A comprehensive literature search across three electronic databases yielded a total of 2,205 unique records. After removing duplicates, 1,448 records were screened by title and abstract, of which 158 were selected for full-text retrieval. A total of 119 full-text articles were assessed for eligibility, and 16 articles were included in the final review, representing the authorisation pathways for COVID-19 vaccines in nine countries/territories (USA, EU, Japan, Canada, China, UK, Mexico, Brazil, and India).

In addition, 43 official regulatory documents related to COVID-19 vaccine authorisation were retrieved from NRA websites and included in the review. Among the 23 initially targeted countries, three (Chile, Israel, and Russia) were excluded due to insufficient or inaccessible information regarding their authorisation pathways.

**Figure 1.**
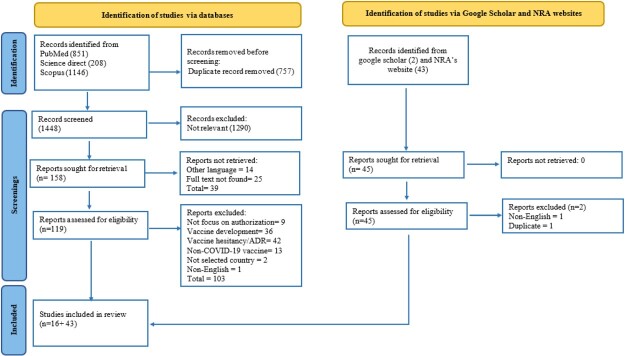
PRISMA-ScR flow diagram.

In total, data from 20 countries were included in the review, comprising 12 high-income, 5 upper middle-income, and 3 lower middle-income countries. Notably, 30 out of 43 regulatory documents were newly issued in response to the COVID-19 pandemic, while the remaining 13 had been in effect prior to the outbreak. The newly introduced regulations aimed to clarify and update emergency use authorisation processes for COVID-19 vaccines and other related medical products. The study selection process is presented in Figure 1. Further details on regulatory frameworks and country-specific descriptions are provided in Table 2.

**Table 2.** Description of included countries, law-regulation-guidance related to COVID-19 vaccine regulation.

Country	NRA*	Economic level**	Law, regulation, guidance related to COVID-19	Vaccines *Approval date*	Confirmed COVID-19 cases/Million population***
AUS	TGA	HIC	1. Therapeutic Good Act 1989 and Therapeutic Good Regulation 1990 2. Provisional registration process for prescription medicines with provisional determination Version 1.1, Aug 2018	COMIRNATY *25 Jan 2021* VAXZEVRIA *15 Feb 2021*	9
CAN	HC	HIC	1. Interim Order Respecting the Importation, Sale, and Advertising of Drugs for Use in Relation to COVID-19 (ISAD IO) (16 Sep 2020) 2. Amendment Food and Drug Regulations (16 Sep 2021) 3. Information and application requirements for drugs authorized under the Interim Order: Guidance document (16 Nov 2020)	COMIRNATY *9 Dec 2020* VAXZEVRIA *26 Feb 2021* SPIKEVAX *26 Feb 2021*	5,931
CHE	SWISS MEDIC	HIC	1. Ordinance 3 of 19 June 2020 on Measures to Combat the Coronavirus (SR 818.101.24) 2. Guidance document, Authorisation procedures for Covid-19 medicinal products during a pandemic HMV4 version 3. 0 3 Sep 2020 focused on COVID-19 (revised from 1st version 19 Jan 2012)	COMIRNATY *19 Dec 2020* SPIKEVAX *12 Jan 2021* JCOVDEN *22 Mar 2021*	3,817
DEU	PEI	HIC	1. Centralised procedure (Regulation 726/2004) by EMA	COMIRNATY *21 Dec 2020* SPIKEVAX *6 Jan 2021* VAXZEVRIA *29 Jan 2021*	695
ESP	AEMPS	HIC	1. Centralised procedure (Regulation 726/2004) by EMA	COMIRNATY *21 Dec 2020* SPIKEVAX *6 Jan 2021* VAXZEVRIA *29 Jan 2021*	8,062
EU	EMA	HIC	1. Article 14-A of Regulation (EC) No 726/2004 of the European Parliament and of the Council of 31 March 2004 2. Commission Regulation (EC) No 507/2006 of 29 March 2006 on the conditional marketing authorisation for medicinal products for human use falling within the scope of Regulation (EC) No 726/2004 3. Guideline on clinical evaluation of vaccines 26 Apr 2018 (EMEA/CHMP/VWP/164653/05 Rev. 1) 4. EMA considerations on COVID-19 vaccine approval version dated 16 Nov 2020	COMIRNATY *21 Dec 2020* SPIKEVAX *6 Jan 2021* VAXZEVRIA *29 Jan 2021*	134,366
GBR	MHRA	HIC	1. Temporary Authorisation under Regulation 174 (R174) of the Human Medicines Regulations 2012 2. Guidance on Conditional Marketing Authorisations, exceptional circumstances Marketing Authorisations and national scientific advice (Effective date 1 Jan 2021)	COMIRNATY *2 Dec 2020* VAXZEVRIA *30 Dec 2020* SPIKEVAX *8 Jan 2021*	14,594.00
JPN	PMDA	HIC	1. Article 14-3, Paragraph 1 in the Act on Securing Quality, Efficacy, and Safety of Products Including Pharmaceuticals and Medical Devices (Act No. 145 of 1960) 2. Principles for the Evaluation of Vaccines Against the Novel Coronavirus SARS-COV-2 version date 2 Sep 2020	COMIRNATY *14 Feb 2021* SPIKEVAX *21 May 2021* VAXZEVRIA *21 May 2021*	2,053
KOR	MFDS	HIC	1. Pharmaceutical Affairs Act (PAA) 1960 and amended in 2002 and 2020.	VAXZEVRIA *10 Feb 2021* COMIRNATY *5 Mar 2021*	471
SGP	HSA	HIC	1. Sub-regulations 60A (4) and (5)(b) of the Health Product (Therapeutic Products) Regulations. Amendment 2020. 2. Guidance note on Pandemic Special Access Route (PSAR) for Supply of Emergency Therapeutic Products; 1 Dec 2020 and Annex A, Jul 2021 3. Special Access Route for unauthorised COVID-19 vaccines May 2021	COMIRNATY *14 Dec 2020* SPIKEVAX *3 Feb 2021* CORONAVAC *23 Oct 2021*	58,297
TWN	Taiwan FDA	HIC	1. Article 48-2 of the Pharmaceutical Affairs Act import the specific drug as a special case (Amendment 31 Jan 2018) 2. Regulations for Approval of Specific Medical Products’ Manufacturing or Importing (Amend dated 27 Jul 2022) by Ministry of Health and Welfare 3. The standards for granting EUA for COVID-19 vaccines to prevent COVID-19 public health emergency, June 2021 (non-English document)	VAXZEVRIA *20 Feb 2021* SPIKEVAX *22 Apr 2021* COMIRNATY *3 Aug 2021* MEDIGEN *30 Jul 2021*	8
USA	FDA	HIC	1. Regulations in 21 CFR Part 312 and licensing regulations in 21 CFR Part 601 2. Development and Licensure of Vaccines to Prevent COVID-19 Guidance for Industry, Jun 2020 3. Process for Making Available Guidance Documents Related to Coronavirus Disease 2019. Federal Register /Vol. 85, No. 58 / Mar, 2020 /Notices 4. Emergency Use Authorisation for Vaccines to Prevent COVID-19: Guidance for Industry May 2021, and Mar 2022	COMIRNATY *11 Dec 2020* SPIKEVAX *18 Dec 2020* JCOVDEN *27 Feb2021*	164,231
BRA	ANVISA	UMIC	1. Resolution of the Collegiate Board-RDC No. 444 of 12/10/2020: Establishes the temporary authorisation for the emergency use, on an experimental basis, of Covid-19 vaccines to face the public health emergency of national importance resulting from the outbreak of the new coronavirus (SARS-CoV-2) (revoked) 2. Guide on the minimum requirements for submitting a temporary authoriation request for emergency use, on an experimental basis, of Covid-19 vaccines (RESOLUÇÃO DA DIRETORIA COLEGIADA – RDC N° 55, DE 16 DE DEZEMBRO DE 2010 (Guide No 42 version 1 of 12/2/2020) 3. Legislation: Resolution RDC no.475 for Emergency Use Authorisation, 10 Mar 2021	VAXZEVRIA *17 Feb 2021* CORONAVAC *17 Feb 2021* COMIRNATY *25 Feb 2021*	35,678
CHN	NMPA	UMIC	1. ‘Drug Registration Rules’ (‘DRR’) (SAMR Order No.27) the State Administration for Market Regulation (SAMR) 30 Mar 2020 2. Revised Drug Registration Measures, July 2020	CORONAVAC *23 Aug 2020* COVILO *31 Dec 2020*	17
MEX	COFEPRIS	UMIC	1. Government of Mexico, ‘Agreement,’ 19 March 2021 The National Vaccination Policy against the SARS-CoV-2 Virus for the Prevention of COVID-19 in Mexico	COMIRNATY *Jan 2021^24^*	22
MYS	NPRA	UMIC	1. Regulation 8 of Control of Drug and Cosmetics Regulations 1984 2. Guidance and Requirements on Conditional Registration for Pharmaceutical Products During Disaster Rev 1.0: July 2021	COMIRNATY *8 Jan 2021* VAXZEVRIA *2 Mar 2021*	291
THA	Thai FDA	UMIC	1. Drug Act B.E. 2510 (AC 1967) and its amendment section 13 (5) 2. Thai FDA Notification for conditional approval for emergency use of medicinal product version dated 24 Jul 2020	VAXZEVRIA *Jan 2021* CORONAVAC *22 Feb 2021* JCOVDEN *25 Mar 2021* COVILO *28 May 2021*	11
IDN	BPOM	LMIC	1. Pelaksanaan Persetujuan Penggunaan Darurat (Implementation of Emergency Use Authorisation) issued on 9 Nov 2020	CORONAVAC *13 Jan 2021* SPIKEVAX *9 Mar 2021*	5,253
IND	CDSCO	LMIC	1. Draft regulatory guidelines for development of vaccines with special consideration for COVID-19 vaccines. (20 Sep 2020) 2. Guidance for approval of COVID-19 vaccines in India for restricted use in emergency which are already approved for restricted use by US FDA, EMA, UK MHRA, PMDA or which are listed in WHO EUL (15 Apr 2021)	COVISHIELD *27 Jan 2021* COVAXIN *27 Jan 2021*	40,709
PHL	FDA	LMIC	1. Executive Order (EO) No. 121 entitled ‘Granting Authority to the Director General of the Food and Drug Administration to Issue Emergency Use Authorisation (EUA) for COVID-19 Drugs and Vaccines (12 Dec 2020)’ 2. FDA Circular No. 2020-036 || Guidelines on the Issuance of Emergency Use Authorisation for Drugs and Vaccines for COVID-19 (14 Dec 2020)	COMIRNATY *14 Jan 2021* VAXZEVRIA *28 Jan 2021*	1,573

* NRAs’ abbreviation: ANVISA = Agência Nacional de Vigilância Sanitária; AEMP = Agencia Española de Medicamentos y Productos Sanitarios; BPOM = Badan Pengawas Obat dan Makanan; CDSCO = Center Drug Standard Control Organization; COFEPRIS = Cofepris-the Federal Commission for Protection against Health Risks; EMA = European Medicines Agency; FDA = Food and Drug Administration; HC = Health Canada; HSA = Health Sciences Authority; ISP = Instituto de Salud Pública de Chile; MFDS = Ministry of Food and Drug Safety; MHRA = the Medicines and Healthcare products Regulatory Agency; NMPA = National Medical Products Administration; NPRA = National Pharmaceutical Regulatory Agency; PMDA = Pharmaceuticals and Medical Devices Agency; PEI = The Paul-Ehrlich-Institute; TGA = Therapeutic Goods Administration; TWN = Taiwan Food and Drug Administration; Swiss Medic = Swiss Agency for Therapeutic Products.

** Economy level classification by the World Bank: HIC = high-income country; UMIC = upper middle-income country; LMIC = lower middle-income country.

*** Number of confirmed COVID-19 cases (as of 30 Nov 2020) https://ourworldindata.org/covid-cases.

#### Authorisation pathway and product requirement under each authorisation pathway

From 20 countries, 24 regulatory pathways were identified. Three unique types of regulatory pathways; ‘emergency use authorization’ (EUA) (*n* = 10), ‘conditional marketing authorization’ (CMA) (*n* = 8), and ‘temporary authorization’ (TA) (*n* = 6) were classified (please see Table 3). Sixteen countries utilised one regulatory pathway while four countries utilised two regulatory pathways. CHN approved CORONAVAC, the first locally developed vaccine, under EUA and specified that this vaccine was for frontline workers (‘National Medical Products Administration. Official: Emergency use of coronavirus vaccines authorised,’ 2020). The National Medicinal Products Administration (NMPA) subsequently switched to the CMA pathway for other COVID-19 vaccines (The National Medical Products Administration, 2021).

**Table 3.** Eligibility criteria for product approval requirement.

Country/Economic level		Regulatory pathway/Name of the pathway		Indicated criteria for certain regulatory pathway				
				GMP compliance	Benefit-risk balance	No alternative product (unmet need)	On-going evidence submission commitment	Previously approved in other countries
JAP	HIC	Emergency Use Authorization (EUA)	Special approval for emergency use^existing^	✓	✗	✓	✓	✓
TWN			EUA^existing^	✓	✓	✓	✓	✗
USA			EUA^existing^	✓	✓	✓	✓	✗
BRA	UMIC		Emergency Use Approval^new^	✓	✓	✓	✓	✓
CHN			Emergency authorization^new^	✗	✗	✗	✗	✗
MYS			EUA^new^	✓	✓	✓	✓	✓
MEX			EUA^new^	✓	✓	✓	✓	✓
IDN	LMIC		EUA^new^	✓	✓	✗	✗	✗
IND			Emergency approval^new^	✗	✗	✗	✓	✓
PHP			EUA^new^	✓	✓	✓	✓	✓
**% of criteria used**				80%	70%	70%	80%	60%
AUS	HIC	Conditional marketing authorization (CMA)	Provisional Approval^existing^	✓	✓	✓	✓	✗
DEU			CMA^existing^	✓	✓	✓	✓	✗
ESP			CMA ^existing^	✓	✓	✓	✓	✗
KOR			CMA^existing^	✓	✓	✓	✓	✓
GBR			CMA^existing^	✓	✓	✓	✓	✗
EU			CMA^existing^	✓	✓	✓	✓	✗
CHN	UMIC		Conditional Approval^existing^	✓	✓	✓	✓	✗
THA			Conditional Approval for EUA^new^	✓	✓	✓	✓	✓
**% of criteria used**				100%	100%	100%	100%	25%
CAN	HIC	Temporary authorization (TA)	Interim Order^new^	✓	✓	✓	✗	✓
CAN			Authorization under the Food and Drug Regulation 2021^new^	✓	✓	✓	✗	✓
CHE			Temporary Approval^existing^	✓	✓	✓	✓	✗
GBR			Temporary authorization^new^	✓	✓	✓	✓	✗
SGP			Pandemic special access route under interim authorization^new^	✓	✓	✓	✓	✗
SGP			Special access route^existing^	✓	✓	✗	✗	✗
**% of criteria used**				100%	100%	83%	50%	33%
**Overall**				92%	86%	83%	75%	42%

Note: – Meaning of indicated criteria: ✓ =  clearly indicated, ✓ =  indirectly indicated, ✗ = not indicated.

Abbreviations: GMP = Good Manufacturing Practice, MA = marketing authorisation, EUA = emergency use authorisation.

Country abbreviation: AUS = Australia, DEU = Germany, ESP = Spain, KOR = South Korea, CHE  = Switzerland, GBR = United Kingdom, EU = European Union, BRA = Brazil, CAN = Canada, CHN = China, THA = Thailand, JAP = Japan, TWN = Republic of China (Taiwan), USA = United States of America, MYS = Malaysia, MEX = Mexico, IDN = Indonesia, IND = India, PHP = The Philippines, SGP = Singapore.

New = newly created regulatory pathway, existing = existing regulatory pathway.

Canada and the United Kingdom utilised interim order (Govenment of Canada, 2020) and temporary authorisation (Medicines & Healthcare products Regulatory Agency (MHRA), 2020) at first, but later on switched to authorisation under the Food and Drug Regulation 2021 and CMA, respectively. Singapore had two parallel pathways, a pandemic special access route under interim authorisation and a special access route. The first pathway was used by HSA for vaccines provided by the government, but the latter was open for hospitals to import COVID-19 vaccines outside the government programme.

Regarding the requirement under each regulatory pathway, it was found that compliance with the Good Manufacturing Practice (GMP) was commonly specified by most countries (92%), followed by benefit-risk evaluation (86%), and no alternative product (83%). Countries that utilised ‘CMA’ pathway usually included the criteria regarding the commitment to submit on-going evidence, while countries which utilised ‘EUA’ pathway were likely to include previous approval in other countries in their criteria. Requirements other than those previously mentioned included (1) being innovative product (CAN, SGP, CHE, AUS, JAP, TWN), (2) new indication of existing products (AUS, CAN), and (3) specified target vaccine users (CHN, THA).

#### Supporting strategies and regulatory network

Based on what was written in the 24 regulatory documents, this study specified six facilitating strategies (please see Table 4). The two most common strategies specified in the regulatory document were ‘rolling submission’ (85%) and ‘pre-submission meeting’ (85%). Rolling submission allowed MAHs to gradually submit the required evidence e.g. safety and efficacy from on-going clinical trials. Through the rolling submission strategy, NRAs expedited access to COVID-19 vaccines during the intense pandemic by making approval decisions based on preliminary information.

**Table 4.** Supporting strategies and international regulatory network.

Country/regulatory pathway			Strategic regulatory tools						Regulatory network		References
			Rolling submission/Review	Pre-submission meeting	Reliance	Recognition	Priority designation	Exemptions	Access Consortium	ICMRA member	
JPN	Emergency Use Authorisation (EUA)	Special approval for emergency use	✓	✓					✓	✓	(Pharmaceuticals and Medical Devices Agency (PMDA), 2020a, 2020b, 2021)
USA		EUA	✓	✓			✓	✓		✓	(U.S Food and Drug Administation, 2022)
TWN		EUA	✓	✓	✓		✓				(Taiwan Food and Drug Administration, 2020)
BRA		Emergency use approval	✓	✓	✓^a^					✓	(Fonseca et al., 2021)
CHN		Emergency use approval	✓							✓	(‘National Medical Products Administration. Official: Emergency use of coronavirus vaccines authorised,’ 2020)
MEX		EUA	✓	✓	✓^b^					✓	(Padron-Regalado & Medina-Rivero, 2022)
MYS		Emergency approval	✓	✓	✓^c^	✓^c^	✓				(*Bahagian Regulatori Farmasi Negara (NPRA). Guidance and Requirements on Conditional Registration for Pharmaceutical Products During Disaster*, 2022)
IDN		EUA			✓^b^						
IND		EUA	✓	✓	✓^d^					✓	(Central Drugs Standard Control Organisation (CDSCO), 2021; Central Drugs Standard Control Organisation (CDSCO). COVID-19 vaccines approved in the country, 2022; Dinda et al., 2020)
PHP		EUA			✓^e^	✓^e^					(Food and Drug Administation Philippines, 2020; Food and Drug Administration Philippines, 2022)
AUS	Conditional Marketing Authorisation (CMA)	Provisional approval pathway	✓	✓			✓		✓	✓	(Therapeutic Goods Administration, 2021)
DEU		Conditional MA		✓		✓				✓	(Paul Ehrlich Institute. COVID-19 Vaccines, 2020)
ESP		Conditional MA	✓	✓		✓				✓	(Agencia Española de Medicamentos y Productos Sanitarios, 2022)
EU		Conditional MA	✓	✓			✓	✓		✓	(*European Medicine Agency (EMA). COVID-19 vaccines*)
GBR		Conditional MA	✓	✓					✓	✓	(Medicines & Healthcare products Regulatory Agency (MHRA), 2020)
KOR		Conditional MA	✓	✓						✓	(Ministry of Food and Drug Safety (MFDS))
CHN		Conditional Approval	✓	✓						✓	(‘National Medical Products Administration. Official: Emergency use of coronavirus vaccines authorised,’ 2020)
THA		Conditional Approval for EUA	✓*	✓	✓^f^		✓	✓			(Thai Food and Drug Administration, 2021)
CAN	Temporary Authorisation (TA)	Interim Order	✓	✓			✓	✓	✓	✓	(Lythgoe & Middleton, 2021)
CAN		Authorised under the Food and Drug Regulations 2021	✓					✓		✓	(Govenment of Canada, 2020)
CHE		Temporary authorisation	✓	✓					✓	✓	(Swiss Agency for Therapeutic Products (Swissmedic), 2022; Swiss Agency for Therapeutic Products (Swissmedic). Current status of authorisations for combating COVID-19, 2022)
GBR		Interim Authorisation	✓						✓	✓	(Abbas & Babar, 2021; Government of the United Kingdom, 2020; Mahase, 2020)
SGN		Special Access route	✓	✓	✓^g^				✓	✓	(Health Singapore Authority, 2021b)
SGN		Pandemic Special Access Route	✓	✓	✓^g^				✓	✓	(Health Singapore Authority, 2021a)

Note: ✓ = clearly mentioned, ✓ = not clearly mentioned, blank = not directly mentioned.

a = BRA reliance with US FDA, EMA, PMDA and NMPA, b = Reliance country not specified, c = Malaysia reliance or recognition with US FDA, EMA or WHO, d = India reliance with US FDA, EMA, UK MHRA, PMDA or WHO, e = Philippines reliance with the US FDA, TGA, EMA, HSA, PMDA, MFDS, MHRA, Swiss Medic, Health Canada and recognition with the WHO, f = Thailand reliance with US FDA, EMA, PMDA, TGA or WHO, g = Singapore reliance with US FDA, EMA, TGA, HC and MHRA, * = Rolling submission was not clearly stated in the official document, but actually conducted in real practice.

The pre-submission meeting is another common strategy used in the regular drug registration process. It is usually initiated by the MAHs to resolve scientific issues related to the product development process and to seek mutual understanding for which evidence needs to be generated to support marketing authorisation. Note that both rolling submission and the pre-submission meeting are considered a common strategy. Countries with existing pathways to respond to emergency or pandemic situations have already implemented both strategies in their routine drug approval pathway. Thus, not clearly mentioning rolling submission and pre-submission meeting did not indicate that the two strategies were not implemented.

Reliance was a strategy clearly mentioned among 10 countries. Eight of them, except for Singapore and the Republic of China, were non-high-income countries. In general, reliance means the NRA of one country relies on the assessment performed by other trusted NRAs to support its own approval decision. The countries that were declared as references for reliance were USA (*n* = 6), EU (*n* = 6), Japan (*n* = 4), WHO (*n* = 4), Australia (*n* = 3), United Kingdom (*n* = 3), Canada (*n* = 2) Singapore (*n* = 1), Switzerland (*n* = 1), and South Korea (*n* = 1).

Recognition is another strategy that shortens the approval process much faster than the reliance strategy. When recognition is implemented, it means that the NRA of one country can adopt the approval decision of the trusted NRAs without an internal process. Malaysia and Philippines clearly declared the use of the recognition strategy. Malaysia recognises the EU, USA and WHO, while the Philippines recognises the WHO. Germany and Spain did not clearly mention recognition, but as EU member states, they recognise the EMA.

Besides recognition and reliance strategies, the NRA of each country may participate in a regulatory network such as the ‘Australia-Canada-Singapore-Switzerland-United Kingdom Consortium-Access Consortium’ (Government of Canada, 2020) or the ‘International Coalition of Medicines Regulatory Authorities (ICMRA)’ (International Coalition of Medicines Regulatory Authorities, 2022). The aim of the Access Consortium is to maximise collaboration by sharing expertise and resources among member countries and ensure consistency and current approach to make approval decisions for the fast-moving pace of emergence and new technology therapeutic products.

ICMRA is another international coalition encompassing 22 member countries and 15 associate member countries. The WHO serves as an observer in ICMRA. ICMRA aims to support global dialogue and facilitate the exchange of reliable information and resources among NRAs.

Priority designation is a strategy that exists in seven NRAs; Australia, Canada, USA, Malaysia, Republic of China, and Thailand and the EU. A priority designation is used to specify which dossier needs to be considered in a timely manner under an appropriate special regulatory pathway.

Exemptions is a strategy clearly reported in the regulatory pathways in Canada, EU and USA. In Canada, the Interim Order notably exempted the MAHs from the disclosure of some data e.g. case report forms (CRFs), consent forms, blinding and randomisation procedures, statistical analysis plan and trial results which can be accessed via Portal (Edmonds et al., 2020). For the EU, exemptions were allowed for labelling, multi-languages requirement and shelf-life information (*European Medicine Agency (EMA). Questions and answers on labelling flexibilities for COVID19 vaccines*, 2022). In the USA, the Investigational New Drug (IND) exemption for the COVID-19 vaccines can be obtained from the FDA. In addition, the MAHs can submit their dossier with the exemption of the administration part under the EUA. The exemption was not clearly mentioned by the Thai FDA, although the MAHs were exempted from vaccine retainment in Thailand given that the MAHs retain vaccine samples in the vaccine originating country.

#### Safety-related aspect

Additional safety requirement aspects were captured. It was found that most countries (*n* = 18) stated that the MAHs should conduct post authorisation pharmacovigilance. As safety data from clinical trials was limited, it was insufficient to detect rare adverse events. In addition, the study time was not long enough to detect adverse effects that occur later. Risk management plan (RMP) and pharmacovigilance activities for COVID-19 vaccines were highly encouraged. In addition, 10 countries with different pathways clearly specified that MAHs must obtain lot release approval before vaccine deployment in the countries. This aligns with a study performed by WHO (Khadem Broojerdi et al., 2021), which indicated that lot release may not be necessary for regulatory preparedness to approve Medical Products during Public Health Emergencies. The results from this study further indicated that the necessity for lot release as a prerequisite for approving COVID-19 vaccines during the pandemic could be linked to one specific regulatory pathway, the EUA. It was found that the EUA is less likely to require lot release, whereas no conclusive evidence suggested a connection between lot release requirements and the CMA and TA.

#### Approval time

Vaccine approval is an important output for the regulatory decision process. Vaccination rollout is directly related to vaccine approval. Under the pandemic situation, it is worth assessing how long it took to review and make an approval decision. The first approved vaccines from 20 countries were specified. This choice was made because this study believed it would accurately depict the efficiency of the approval process during the pandemic. Having the COVID-19 vaccine dossiers submitted to each NRA depended on various factors (e.g. economic, political factors and platform of the vaccine, etc.). This study chose to exclusively compare the first approved and most frequently approved vaccine (COMIRNATY). This approach helped this study avoid potential biases associated with these factors. The submission date and approval date were sought. Details of first approved vaccine, submission date, approval date, and standard review time are shown in Table 5.

**Table 5.** Comparison of the review time for the first COVID-19 vaccine with the standard review time.

Country	Regulatory pathway	1st approved vaccine	Submission date	Approval date	Review time (days)	Standard Review time (days)	x times faster than standard review
GBR	TA	COMIRNATY	1-Oct-20	2-Dec-20	62	210	3.4
CAN	TA	COMIRNATY	10-Sep-20	9-Dec-20	90	210	2.3
MEX	EUA	COMIRNATY	N/A	11-Dec-20		240/60*	
USA	EUA	COMIRNATY	20-Nov-20	11-Dec-20	21	210	10.0
SGP	TA	COMIRNATY	4-Dec-20	14-Dec-20	10	270	27.0
CHE	TA	COMIRNATY	16-Oct-20	19-Dec-20	64	480	7.5
EU	CMA	COMIRNATY	30-Nov-20	21-Dec-20	21	210	10.0
DEU	CMA	COMIRNATY	N/A	Same as EU		Same as EU	
ESP	CMA	COMIRNATY	N/A	Same as EU		Same as EU	
CHN	EUA	CORONAVAC	N/A	31-Dec-20		420	
IND	EUA	COVISHILED	N/A	3-Jan-21		270	
IND	EUA	COVAXIN	N/A	3-Jan-21		270	
MYS	EUA	COMIRNATY	14-Dec-20	8-Jan-21	25	245/120*	9.8/4.8
IDN	EUA	CORONAVAC	N/A	13-Jan-21		300	
PHP	EUA	COMIRNATY	5-Dec-20	14-Jan-21	21	180	8.6
BRA	EUA	VAXZEVRIA	N/A	17-Jan-21		365/120^§^	
BRA	EUA	CORONAVAC	N/A	17-Jan-21		365/120^§^	
THA	CMA	VAXZEVRIA	30-Dec-20	20-Jan-21	21	280	13.3
AUS	CMA	COMIRNATY	2-Nov-20	25-Jan-21	84	255	3.0
KOR	CMA	VAXZEVRIA	4-Jan-21	10-Feb-21	36	180	5.0
JAP	EUA	COMIRNATY	18-Dec-20	14-Feb-21	58	240	4.1
TWN	EUA	VAXZEVRIA	N/A	20-Feb-21		360	

Note * = standard review time for COVID-19 vaccine.

§ = standard review time for priority designation medicine.

N/A = data not available.

From 20 countries, the first vaccines approved were COMIRNATY (*n* = 13), VAXZEVRIA (*n* = 4), CORONAVAC (*n* = 3), COVISHIELD (*n* = 1), and COVAXIN (*n* = 1). There were two countries; Brazil and India which approved two vaccines at the same time. In general, standard review time ranges from 210 to 480 days. BRA specified 120 days for the standard review time for priority-designated medicine, while MYS and Mexico specified 120 and 60 days, respectively, for the standard review time for the COVID-19 vaccine. Both the submission and the approval dates were retrievable from 12 countries. It was found that the actual review time for the first COVID-19 vaccine ranged from 10 days to 84 days. The UK spent 62 days reviewing and approving COMIRNATY which was the first approved COVID-19 vaccine in the world. Five countries reviewed and approved their first COVID-19 vaccine within 30 days: Singapore (10 days), the EU, Philippines, Thailand, EU (21 days), and Malaysia (25 days). Overall, it can be said that every country effectively reviewed its first COVID-19 vaccine as the review times were 3–27 times faster than standard review times. Malaysia which specified a new standard approval time for the COVID-19 vaccine at 120 days also approved the vaccine 4.8 times faster than what was specified. When considering the review time for different pathways and strategies, the analysis revealed that among the three pathways for approving the COMIRNATY Vaccine, the EUA pathway exhibited the shortest average review time (39.5 days), followed by CMA (42 days), and TA (62.33 days). However, when recognition or reliance strategies were combined with the basic regulatory pathway, the average review time significantly decreased, for instance, to 15.67 days for EUA with recognition/reliance strategies and to 10 days for TA with a recognition strategy.

## Discussion

Although there were only a few studies exploring the regulatory pathways for COVID-19 vaccines, this scoping review provided more extensive information as it reviewed actual regulatory pathways and covered countries from broader geographic regions and economic levels.

Three regulatory pathways; EUA, followed by CMA, and TA were identified. These pathways were previously mentioned in a review conducted by CIRS during the development of the COVID-19 vaccines in 2020 (Centre for Innovation in Regulatory Science, 2020) and were consistent with a study conducted among countries in Latin America in 2022 (van der Zee et al., 2022).

The three regulatory routes share a common goal, which is to expedite the approval procedure. Considering the eligibility criteria for vaccine approval, the EUA employs a less stringent set of criteria. Consequently, of the three pathways studied, the EUA approach involved the shortest average review time. Despite the CMA imposing a greater array of prerequisites, the average review time closely resembled that of the EUA. This could potentially be elucidated by the fact that 7 out of 8 countries that employed the CMA had prior experience with its utilisation. This scoping review also found that countries that utilised CMA required MAHs to fulfil the requirements; (1) GMP compliance, (2) Benefit-risk balance, (3) No alternative product (unmet need), and (4) On-going evidence submission commitment more than other countries utilising EUA and TA. This assumption was confirmed by the evidence from China and the UK. Initially, China used a new EUA, while the UK utilised temporary authorisation pathway to handle the pandemic situation. Later, both countries switched to the conditional marketing authorisation pathway which was an existing pathway in those countries.

The three regulatory pathways were not new, especially among countries with stringent NRAs. US FDA approved anthrax vaccine in 2005 and TAMIFLU in 2010 using EUA (U.S Food and Drug Administation [n.d.]. Emergency Use Authorisation – Archived Information). The EMA approved Pazopanib in 2018 by CMA (European Medicine Agency, 2023). NRAs of the upper middle-income and lower middle-income countries were not well prepared for the global pandemic as they did not have an existing regulatory authorisation pathway to handle the situation. These countries adopted either the EUA or TA. The pandemic urged the upper middle-income and lower middle-income countries to establish a system they normally did not have.

High-income countries usually rely on a network of the same level of NRAs e.g. ICMRA. On the other hand, the upper middle-income and lower middle-income countries relied on stringent NRAs. Reliance and/or recognition strategies allowed those countries to utilise evidence previously evaluated from reliable reference countries to shorten the approval process.

COVID-19 vaccines were speedily authorised and rolled out countrywide and worldwide. Post-approval pharmacovigilance activities played a critical role in informing the regulator of all missing safety profiles during the early product research and development phase.

The benefit-risk ratio of products approved during the severe global pandemic might have a lower positive margin with fewer comprehensive evidence when compared to approval under the standard regulatory procedure. Thus, the NRAs should grant a full marketing authorisation once all required evidence has been submitted.

This scoping review focused only on the regulatory approval process. A future area of research should pay attention to how NRAs actually evaluated the dossiers. In addition, a future study should also emphasise on the pharmacovigilance of COVID-19. Some limitations are worth nothing. First, this scoping review relied solely on publicly available information and did not include triangulation through stakeholder interviews from the studied countries. Second, information in published documents may not have clearly described regulatory criteria such as GMP compliance or benefit-risk assessment, or common regulatory strategies such as pre-submission meetings. These elements were included in Tables 3 and 4 using specific symbols to indicate presence or absence. Third, the study focused exclusively on COVID-19 vaccines, excluding other related medical products such as therapeutics and diagnostic tools. Fourth, although major scientific databases such as PubMed, Scopus, and ScienceDirect were searched, relevant publications were limited. Accessing regulatory data on official NRA websites also posed challenges due to limited search functions, requiring the use of advanced Google search techniques to retrieve relevant documents. Lastly, the review was limited to English and Thai language sources. Important documents published in other languages may have been excluded, potentially leading to language bias.

## Conclusion

EUA, CMA and TA were the three commonly used pathways among 20 studied countries. These regulatory pathways together with pre-submission meeting, rolling submission, priority designation and exemption were utilised. Networking of NRAs was common among high-income countries, while reliance and recognition were common among non-high-income countries. Various countries efficiently approved COVID-19 vaccines in significantly shorter timeframes compared to standard review times. EUA has yielded the quickest approval, followed closely by CMA. Employing reliance and/or recognition strategies in conjunction with the regular pathway can further expedite the approval process.

Amid the global pandemic, many countries established new regulatory pathways for the first time. It is an appropriate time to review and assess whether the chosen approaches are appropriate, effective, and require adjustments. This study’s findings, which shed light on best practices, can guide decisions aimed at enhancing the regulatory process in future pandemic scenarios.

## Authors’ contributions

SS led the study design, conducted data collection and analysis, performed data charting, and drafted the initial manuscript. PL contributed to the conceptualisation and study design, assisted with screening and data extraction, helped structure the manuscript, and participated in its revision. ON contributed to the conceptualisation and study design, supported data validation and manuscript structuring, and was involved in revising the manuscript. PA supervised the project, supported the conceptualisation and study design, supported data validation, project administration, and provided critical review, editing, and final approval of the manuscript.

## Supplementary Material

Supplemental Material - Appendix

## Data Availability

All relevant data are within the manuscript.
