# Supplementary material for: A scoping review of authorisation pathway for COVID-19 vaccines among selected countries
Source: J Pharm Policy Pract. 2025 Jul 2;18(1):2520861. doi: 10.1080/20523211.2025.2520861 (PMC12224729; doi:10.1080/20523211.2025.2520861)
Supplement: Supplemental Material - Appendix [file JPPP_A_2520861_SM9155.docx]

**Appendix I: Search strategy**

**Article title:** A scoping review of authorization pathway for COVID-19 vaccines among selected countries

**Journal name:** Journal of Pharmaceutical Policy and Practice

Searching on date: 22 May 2023

| Database | Search term | Results |
| --- | --- | --- |
| Pubmed | Search: ("Marketing Authorization" OR "Regulatory approval" OR "approval") AND (COVID-19 vaccine OR SARS-CoV-2 Vaccine) Filters: from 2019/12/1 - 2022/12/31  (("Marketing Authorization"[All Fields] OR "Regulatory approval"[All Fields] OR "approval"[All Fields]) AND ("covid 19 vaccines"[Supplementary Concept] OR "covid 19 vaccines"[All Fields] OR "covid 19 vaccine"[All Fields] OR "covid 19 vaccines"[MeSH Terms] OR ("covid 19"[All Fields] AND "vaccines"[All Fields]) OR ("covid 19 vaccines"[Supplementary Concept] OR "covid 19 vaccines"[All Fields] OR "sars cov 2 vaccine"[All Fields] OR "covid 19 vaccines"[MeSH Terms] OR ("covid 19"[All Fields] AND "vaccines"[All Fields])))) AND (2019/12/1:2022/12/31[pdat]) | 851 |
| Scopus | ( TITLE-ABS-KEY ( "Marketing Authorization" OR "Regulatory approval" OR "approval" ) ) AND ( TITLE-ABS-KEY ( "COVID-19 vaccine" OR "SARS-CoV-2 Vaccine" ) ) AND PUBYEAR > 2018 AND PUBYEAR < 2023 | 1146 |
| Science Direct | Year: 2020-2022 Title, abstract, keywords: ("Marketing Authorization" OR "Regulatory approval" OR "approval") AND (COVID-19 vaccine OR SARS-CoV-2 Vaccine) | 208 |
| Google Scholar | ("Marketing Authorization" OR "Regulatory approval" OR "approval") AND (COVID-19 vaccine OR SARS-CoV-2 Vaccine) AND (“Country Name”) | Only the first 50 articles identified from Google Scholar searches were screened. |
| The NRA’s website section on the selected country | ("Marketing Authorization" OR "Regulatory approval" OR "approval") AND (COVID-19 vaccine OR SARS-CoV-2 Vaccine or specific vaccine name) | 43 documents |
